# Targeting the nucleic acid oxidative damage repair enzyme MTH1: a promising therapeutic option

**DOI:** 10.3389/fcell.2024.1334417

**Published:** 2024-01-31

**Authors:** Yifeng Ding, Qingquan Liu

**Affiliations:** Department of Hepatobiliary Surgery, The First Affiliated Hospital of Gannan Medical University, Jiangxi, China

**Keywords:** MTH1, tumor, DNA repair, MTH1 inhibitor, therapeutic strategy

## Abstract

The accumulation of reactive oxygen species (ROS) plays a pivotal role in the development of various diseases, including cancer. Elevated ROS levels cause oxidative stress, resulting in detrimental effects on organisms and enabling tumors to develop adaptive responses. Targeting these enhanced oxidative stress protection mechanisms could offer therapeutic benefits with high specificity, as normal cells exhibit lower dependency on these pathways. MTH1 (mutT homolog 1), a homolog of *Escherichia coli*’s MutT, is crucial in this context. It sanitizes the nucleotide pool, preventing incorporation of oxidized nucleotides, thus safeguarding DNA integrity. This study explores MTH1’s potential as a therapeutic target, particularly in cancer treatment, providing insights into its structure, function, and role in disease progression.

## 1 Introduction

Redox homeostasis is vital for human health, and its imbalance is a key contributor to several major diseases, including type 2 diabetes, atherosclerosis, chronic obstructive pulmonary disease, Alzheimer’s disease, and cancer (Sies, 2015; Zuo et al., 2020). Oxidative stress (OS), defined as a redox imbalance that favors oxidative load, is commonly observed in cancer cells and is increasingly recognized as a hallmark of various cancers (Harris and DeNicola, 2020; Lennicke and Cocheme, 2021). It fosters numerous malignant behaviors in cancer cells (Forman and Zhang, 2021).

Reactive oxygen species (ROS) are key mediators in redox reactions, often regarded as by-products of cellular respiration (Sies et al., 2017). They primarily arise from various enzymatic and chemical reactions, including those involving cyclooxygenase, nicotinamide adenine dinucleotide phosphate (NADPH) oxidase (NOX), xanthine oxidase, lipogenesis, and the iron-catalyzed Fenton reaction (Tejero et al., 2019), These processes generate hydroxyl radicals (-OH), superoxide radicals (O_2_--), and hydrogen peroxide (H_2_O_2_). Typically, ROS in low to moderate concentrations are advantageous, aiding in maintaining intracellular physiological activities and signaling pathways. However, elevated ROS levels can lead to malignant transformation, cellular damage, and even cell death (Sies and Jones, 2020; Hancock and Veal, 2021). Excessive ROS accumulation creates a highly reactive cellular environment, leading to DNA damage through alkylation, deamination, oxo-group modification, methylation, or halogenation of bases. This DNA damage alters base morphology, causing DNA polymerase to misrecognize these bases, leading to their incorrect incorporation into DNA and subsequent disease development.

Over 30 oxidized base products have been identified, with 8-hydroxyguanine (8-oxoG) being the most prevalent. This oxidized form of guanine pairs with cytosine and adenine equally, leading to genetic information and regulatory function alterations (Wood et al., 1990; Moriya et al., 1991; Shibutani et al., 1991). Nucleotides containing 8-hydroxyguanine can corrupt both DNA and RNA, causing replication and transcription errors (Maki and Sekiguchi, 1992). Additionally, 8-hydroxyguanine can be incorporated into RNA as 8-hydroxyguanine nucleoside triphosphate or result from direct oxidation of guanine in RNA (Yanagawa et al., 1992). Mammalian cells have enzymes like Nudix hydrolase 1 (NUDT1, MTH1) that remove these nucleotides from the pool, hydrolyzing 8-oxoGDP to 8-oxoGMP, with MTH1 also acting on 8-oxoGTP. This process helps to prevent intracellular DNA damage, thus reducing cell damage and apoptosis (Ishibashi et al., 2005; Das et al., 2020).

Recent years have seen a surge in research on MTH1, leading to the development of over ten types of MTH1 inhibitors. Despite this progress, the exact roles of MTH1 in diseases and tumors, as well as the therapeutic efficacy of these inhibitors, remain incompletely understood. This paper reviews MTH1’s molecular structure, biological functions, its roles in diseases and tumors, and advances in inhibitor research and novel therapeutic approaches. Additionally, it discusses new therapeutic strategies targeting MTH1 and outlines future research directions in this field.

## 2 Molecular structure and biological function of MTH1

The gene encoding the human-derived MTH1 protein is located on chromosome 7p22 and spans approximately 9 kb, comprising five exons. MTH1 functions by hydrolyzing oxidized deoxyribonucleoside triphosphates, thereby preventing the incorporation of oxidized dNTPs into DNA during replication and maintaining normal cell functionality. Additionally, MTH1 is known for removing methylated purine triphosphates, such as O6-methyl-dGTP, from the nucleotide pool. This unique ability to hydrolyze O6-methyl-dGTP, highly conserved across evolution, is distinctive to MTH1 among human NUDIX enzymes (Jemth et al., 2018).

To elucidate MTH1’s recognition and hydrolysis mechanisms, researchers have resolved its crystal structure using X-ray crystallography (Mishima et al., 2004; Svensson et al., 2011). Within MTH1’s active site, 8-oxo-dGMP adopts an antipodal conformation, engaging in π-stacking interactions with Trp117 and Phe72. Its Watson-Crick face is recognized by Asp119 and Asp120 through hydrogen bonds with the 6-O, 1-NH, and 2-NH2 groups of 8-oxo-dGMP. Asn33, positioned at the purine base’s bottom in 8-oxo-dGMP, forms hydrogen bonds with 2-NH2 and 3-N. Notably, the Trp117Ala mutation impairs MTH1’s ability to hydrolyze 8-oxo-dGTP or 2-OH-dATP. The Asn33Glu mutation entirely inhibits hydrolysis of 8-oxo-dGTP, while the Asn33Ala mutation retains only 14% activity (Mishima et al., 2004).

**FIGURE 1:**
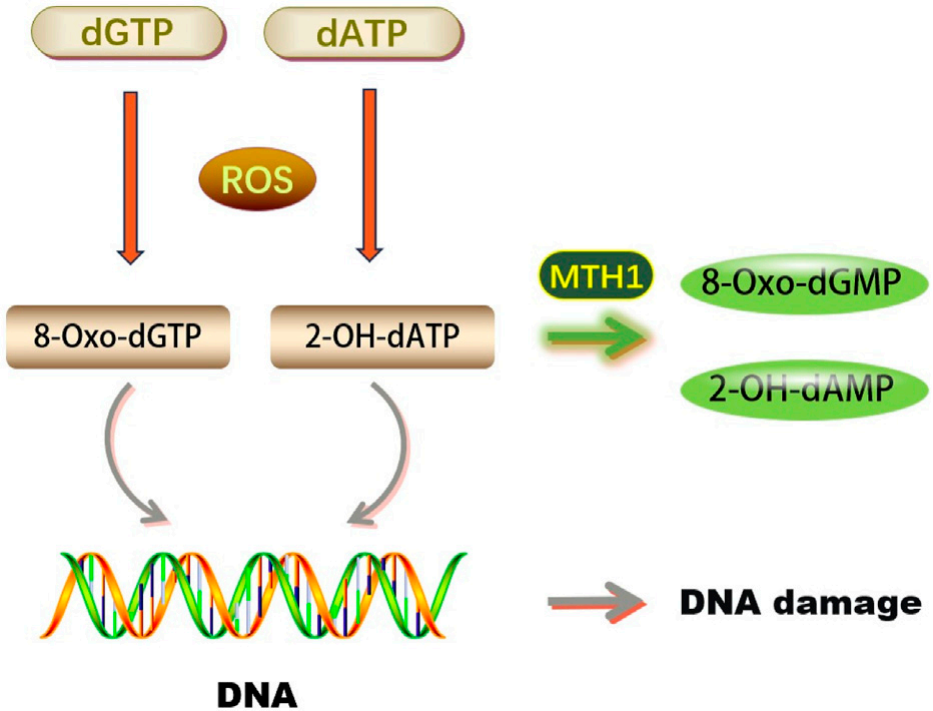
Biological functions of MTH1 (dGTP, Deoxyguanosine triphosphate; dATP, Deoxyadenosine triphosphate; ROS, Reactive Oxygen Species; 8-Oxo-dGTP, 8-oxo-2′-deoxyguanosine-5′-triphosphate; 2-OH-dATP, 2′-Deoxyadenosine-5′-Triphosphate, 2′-OH-Modified; MTH1, MutT Homolog 1; 8-Oxo-dGMP, 8-Oxo-2′-deoxyguanosine-5′-monophosphate; 2-OH-dAMP, 2′-Hydroxy-2′-deoxyadenosine-5′-monophosphate; DNA, Deoxyribonucleic Acid).

DNA molecules are highly susceptible to reactive oxygen species (ROS). Under oxidative stress, ROS can easily damage DNA bases, compromising genome stability. Guanine (G) is particularly vulnerable due to its low redox potential, often being oxidized to 8-oxoG by ROS. This results in 8-oxoG pairing with cytosine (C) in trans and adenine (A) in cis during DNA replication, potentially leading to base pair mutations and impairing DNA’s normal function, as depicted in Figure 1. 8-oxoG is a recognized marker of oxidative DNA damage, indicative of the extent of such damage (Chiorcea-Paquim, 2022).

In both prokaryotic and eukaryotic cells, the removal of 8-oxoG predominantly occurs through the base excision repair pathway, mediated by DNA glycosylase OGG1. In *Escherichia coli*, this process involves a cooperative system known as the ‘GO repair system,’ consisting of DNA glycosylases Mut-M and Mut-Y, along with 8-oxoGTPase Mut-T, to avert mutations induced by 8-oxoG. In human and mouse cells, DNA repair against 8-oxoG-induced mutations is initiated by enzymes including OGG1 and MTH1 (Hirano et al., 2004; Rodrigues et al., 2010; Mahmoud et al., 2022). Consequently, this has spurred extensive research into the functional role of MTH1 in diseases, aiming to develop therapeutic strategies targeting MTH1.

## 3 The role of MTH1 in disease

**FIGURE 2:**
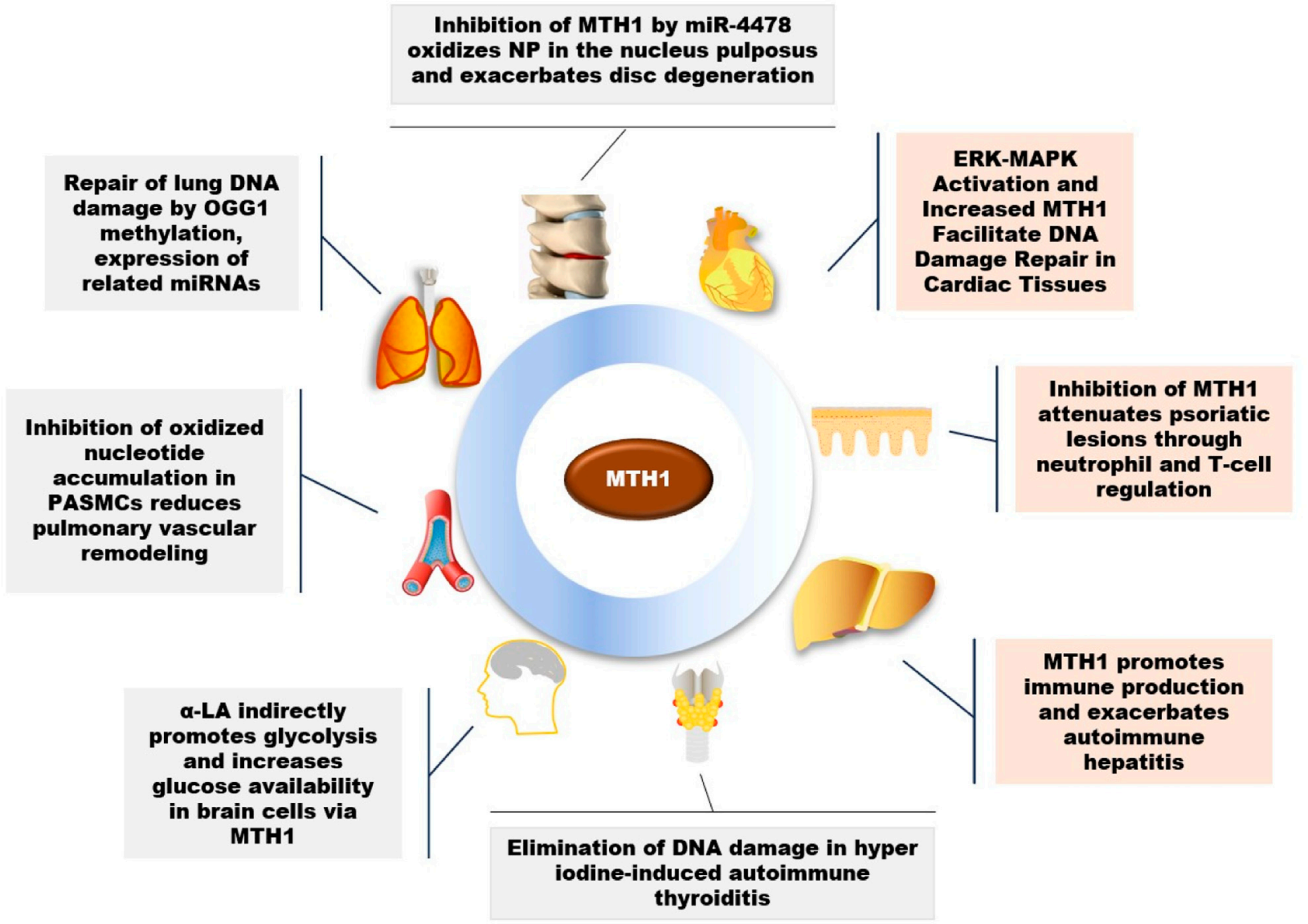
Role of MTH1 in multiple diseases (OGG1, 8-Oxoguanine DNA Glycosylase 1; PASMCs, Pulmonary Artery Smooth Muscle Cells; α-LA, Alpha-Lipoic Acid; NP, Nucleus Pulposus).

A variety of external factors, to varying degrees, impact human health and directly influence cellular activities. MTH1 plays a crucial role in maintaining cellular function and is key in the treatment of various diseases, as illustrated in Figure 2.

### 3.1 MTH1 role in cardiovascular lung disease

MTH1 has been observed to repair DNA damage in lung tissues caused by smoking in young smokers (Smits and Gillespie, 2014). Beyond the impact of smoking, research has also linked fine particulate matter 2.5 (PM2.5), a significant public health hazard known to cause lung diseases, to MTH1. Studies indicate that PM2.5 alters the methylation status of OGG1 DNA and increases the expression of MTH1-associated miRNAs. This change mediates an increase in OGG1 expression and a decrease in MTH1 expression, contributing to lung tissue pathology (Zhao et al., 2022).

Beyond its role in lung tissues, MTH1 plays a crucial part in vascular health, particularly in pulmonary arterial hypertension (PAH), a severe condition marked by abnormally high pulmonary artery pressure and right ventricular failure. A key factor in PAH is the hyperproliferation and anti-apoptosis of pulmonary artery smooth muscle cells (PASMCs), contributing to vascular remodeling. MTH1 has been identified as a pivotal driver of this vascular remodeling in PAH patients and in animal model cells and tissues. Inhibiting MTH1 leads to the accumulation of oxidized nucleotides, resulting in insoluble DNA damage, disrupted cellular bioenergetics, and cell death in PASMCs. Interestingly, the use of the MTH1 inhibitor (S)-Crizotinib has been shown to promote apoptosis in PASMCs, thereby reducing pulmonary vascular remodeling and improving hemodynamics and cardiac function (Vitry et al., 2021).

MTH1 primarily functions to mitigate DNA damage in cells. However, its overexpression in various diseases suggests that inhibiting MTH1 could have therapeutic benefits. RNA oxidation, linked to the onset and progression of many chronic diseases in the elderly, shows a notable connection in heart failure (HF) cases. Elevated levels of 8-oxoGsn in urine and cardiac tissues have been observed in HF, correlating positively with heart failure indicators. This elevation coincides with the activation of the ERK-MAPK pathway and an increase in MTH1 expression. This implies that MTH1 may contribute to the heightened 8-oxoGsn levels in cardiomyocytes, thereby exacerbating damage to these cells (Liu et al., 2019).

### 3.2 MTH1 role in neurological diseases

Oxidative stress is a significant risk factor for Alzheimer’s disease (AD), with notable accumulation of 8-oxo-7,8-dihydroguanine (8-oxoG), an oxidized form of guanine, observed in AD brains. Studies involving AD mouse models have shown that MTH1 and OGG1 reduce oxidized DNA damage in their brains, suggesting a role for MTH1 in preventing the progression of Alzheimer’s disease (Mizuno et al., 2022). Key characteristics of AD include extracellular amyloid plaques, intracellular neurofibrillary tangles (NFTs), and decreased glucose metabolism (Butterfield and Halliwell, 2019). α-Lipoic acid (LA), a natural mitochondrial cofactor, has been shown to mitigate neuronal damage. LA also indirectly enhances glycolysis in brain cells through the activation of proliferator-activated receptor gamma coactivator 1-alpha (PGC-1alpha) and DNA repair enzymes (OGG1/2 and MTH1). This increase in glucose availability is potentially beneficial in treating the AD brain (Zhang et al., 2020a).

### 3.3 Role of MTH1 in skin and immune system diseases

A study on psoriasis revealed elevated MTH1 expression in affected skin. Utilizing a mouse model of psoriasis, it was discovered that inhibiting MTH1 reduced the histopathological features of the disease and normalized neutrophil and T cell levels in the skin and associated lymph nodes (Bivik Eding et al., 2021). Interestingly, in immune system studies, MTH1 is upregulated in rapidly dividing T cells. For instance, *in vitro* stimulated CD3/CD28T cells exhibit higher ROS levels and MTH1 expression compared to resting T cells (Karsten, 2022). Furthermore, research on autoimmune hepatitis showed an overrepresentation of MTH1-positive immune cells in liver samples from patients, correlating with disease severity. This was despite minimal MTH1 expression in hepatocytes, suggesting a significant role of MTH1 in immune cell activity (Chen et al., 2022).

### 3.4 The role of MTH1 in other diseases

Research has also shown that in lumbar disc pathology, miR-4478 may accelerate disc degeneration by increasing oxidative stress in the nucleus pulposus (NP), through the regulation of the target gene MTH1 (Zhang et al., 2023). In studies of autoimmune thyroiditis (AIT), it was observed that hyper iodine (HI) induced inflammation, apoptosis, and DNA damage in mouse thyroid follicular epithelial cells. The overexpression of MTH1 was found to effectively counteract HI-induced cellular inflammation, apoptosis, and DNA damage (Li et al., 2019).

Although MTH1 exhibits similar activities across various diseases, the distinct mechanisms involved in disease pathogenesis result in differing therapeutic effects when targeting MTH1. Therefore, exploring appropriate therapeutic regimens that synergistically treat diseases is necessary. Consequently, MTH1 remains a promising target in the treatment of inflammation-related conditions.

## 4 Biological role of MTH1 in tumors

**FIGURE 3:**
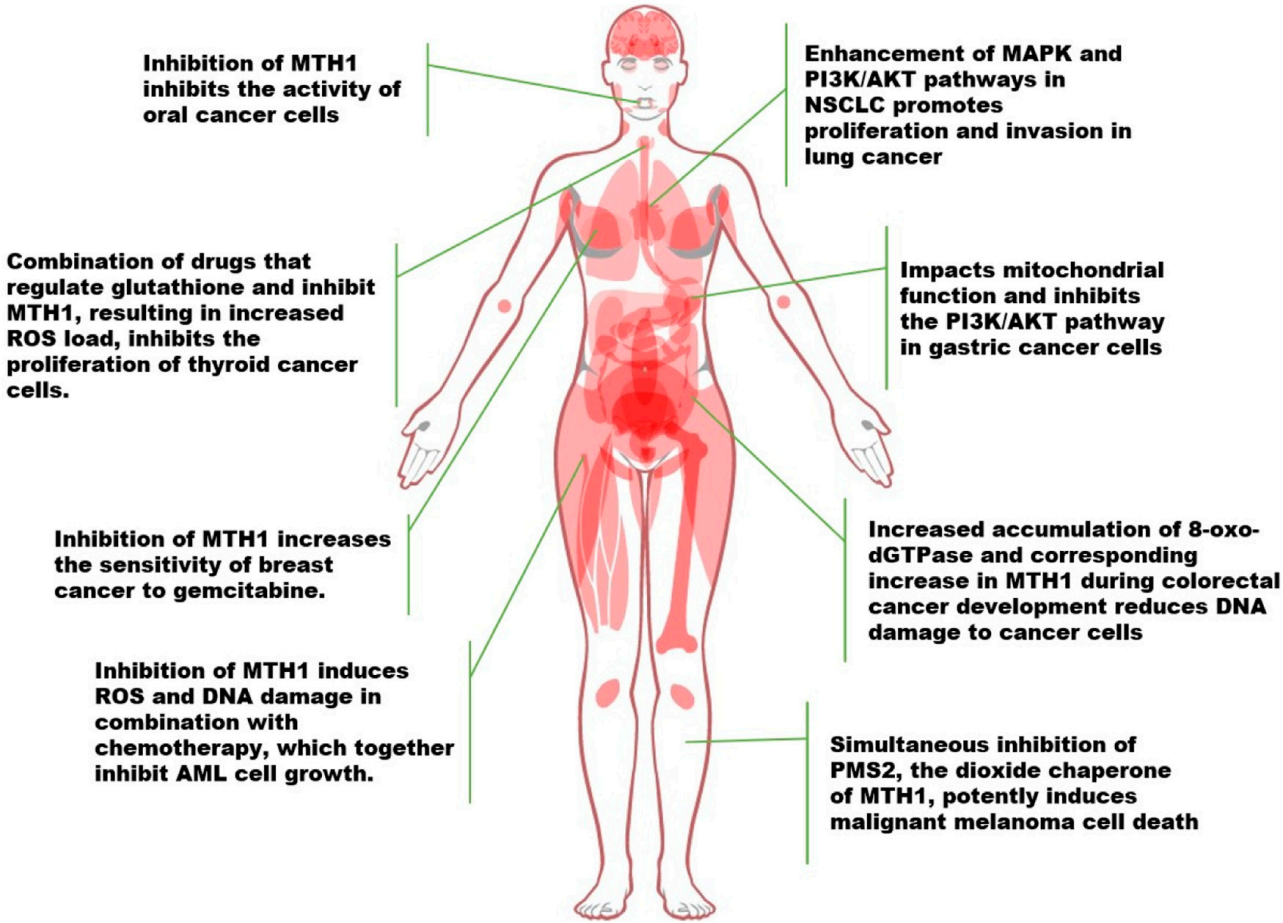
Advances in the study of MTH1 in a variety of tumors (NSCLC, Non-Small Cell Lung Cancer. ROS, Reactive Oxygen Species; 8-oxo-dGTPase, 8-oxo-2′-deoxyguanosine triphosphatase; PMS2, Postmeiotic segregation increased 2).

Tumor cells, characterized by chronic overactivity in mitotic and pro-survival signaling, undergo metabolic changes that generate high levels of oxidants. This results in tumor cells expressing higher levels of MTH1 compared to normal cells. The increased oxidative stress in tumor cells necessitates a reliance on elevated MTH1 expression for maintaining cellular functionality. In numerous tumor types, MTH1 has been observed to directly contribute to tumor growth. Conversely, inhibiting MTH1 synthesis and function can suppress cancer progression, as illustrated in Figure 3.

### 4.1 MTH1 regulates the mitotic process of tumor cells

Researchers developed a chimeric ATP-releasing guanine oxidation probe (ARGO) that merges 8-oxodGTP and ATP to assess MTH1 enzyme activity in various cancers, including colorectal cancer (CRC), non-small cell lung cancer (NSCLC), and pancreatic ductal adenocarcinoma (PDAC), as well as in corresponding normal tissues. They discovered significantly higher levels of MTH1 and 8-oxodGTPase activity in tumor tissues across these cancer types, indicating that MTH1 could serve as a cancer biomarker (McPherson et al., 2019). In exploring how MTH1 activity functions, Patterson et al. employed a method combining experimental synergism quantification with computational pathway-based gene expression analysis, known as the VISAGE strategy. Their findings revealed that inhibiting MTH1 activity, especially when combined with an antimitotic drug like a PLK1 inhibitor, led to the specific eradication of cancer cells. This effect was noted when the MTH1 inhibitor interacted with β-microtubule proteins, affecting microtubule assembly and disrupting mitotic chromosome localization to the spindle’s intermediate band in the presence of the PLK1 inhibitor (Patterson et al., 2019).

### 4.2 Regulation of signaling pathways by MTH1

In a separate study focusing on non-small cell lung cancer (NSCLC), it was discovered that MTH1 not only promotes the progression of NSCLC but also enhances activities in the MAPK pathway and PI3K/AKT in cancer cells. This enhancement led to the induction of epithelial-mesenchymal transition, thus facilitating the proliferative and invasive capabilities of lung cancer cells (Li et al., 2021a). Similarly, in gastric cancer research, the MTH1 inhibitor TH287 was found to exert a range of anticancer effects on gastric cancer cells, impacting mitochondrial function and the PI3K/AKT signaling pathway (Zhan et al., 2020). In the context of rectal cancer, a study investigating MTH1 enzymatic activity, specifically 8-oxo-dGTPase, in human colorectal cancer (CRC) and adjacent cancer-free tissues (CFCF), revealed that the majority of CRC tumors exhibited higher MTH1 activity compared to CFCF. Furthermore, higher 8-oxo-dGTPase activity was associated with a significantly lower likelihood of recurrence-free survival (Bialkowski and Szpila, 2021; Guo et al., 2021).

Other oncogenes, such as NF-κB, MYC, and PI3K, are known to produce ROS, leading to heightened levels of oxidative DNA damage in cells expressing these genes. In tumor cells, MTH1 acts as a protective factor against DNA damage, potentially to avert apoptosis induced by such damage, and may be upregulated in response. This active role of MTH1 has been observed in various cancer types. For instance, in oral cancer treatment research, Shi et al. discovered that counteracting MTH1 activity, in combination with MDR1 siRNA, synergistically inhibited oral cancer cells (Shi et al., 2019). The deletion of MTH1 results in the downregulation of glutathione-dependent oxidative stress (OS) defense system factors. Similarly, studies in thyroid cancer have shown that downregulating MTH1 expression, particularly when combined with drugs regulating the glutathione pool, increases ROS levels in cancer cells. This escalation in ROS impedes the proliferation of thyroid cancer cells and enhances the anticancer efficacy of MTH1 inhibitors (Arczewska et al., 2020; Liao et al., 2020).

### 4.3 MTH1 is regulated by oxidative stress

The development of cancer is influenced by various external factors, including oncogenic stimuli like certain nanoparticles (NPs). These NPs can induce intracellular oxidative stress and cytotoxic effects, leading to DNA damage and the acquisition of an oncogenic phenotype. MTH1 plays a crucial role in mitigating ROS toxicity, which in turn contributes to the proliferation, migration, and invasion of tumor cells, possibly serving as a survival mechanism for them (Barguilla et al., 2020).

Given MTH1’s significant role, non-invasive *in vitro* detection of its expression becomes vital. MTH1, also known as oxidized purine nucleoside triphosphatase, shows promise as a biomarker for monitoring cancer progression and quantifying its involvement in targeted therapies. Notably, its inhibitor TH287, which can be radiolabeled, is detectable *in vitro* assays, especially in brain tumors and other CNS disorders, providing a practical approach for researching MTH1 expression in tumors (Chen et al., 2020). Additionally, research in gliomas has linked MTH1 expression to the hypoxia-inducible factor HIF1α (Zhao et al., 2020).

### 4.4 MTH1 is associated with tumor drug resistance

In human breast cancer cells, particularly MCF7-R, the knockdown of MTH1 activated the expression of the cell cycle protein-dependent kinase inhibitory protein p21. This led to increased sensitivity of MCF7-R cells to gemcitabine, suggesting that silencing the MTH1 gene can enhance the sensitivity of breast cancer cells, potentially serving as a novel adjuvant therapy for targeting drug-resistant tumors (Wen et al., 2021). Similarly, in the study of triple negative breast cancer (TNBC) development, the actin fiber-associated protein 1-antisense RNA1 (AFAP1-AS1) was identified as targeting miR-145 to regulate MTH1 expression, thereby promoting proliferation and invasion. This finding indicates that MTH1 is regulated by upstream genes and elucidates a mechanism in TNBC development (Zhang et al., 2020b).

In the context of acute myeloid leukemia (AML), most patients succumb to primary resistance or relapse due to conventional chemotherapy regimens that induce reactive oxygen species (ROS) and DNA damage. However, combining the MTH1 inhibitor TH1579 with standard chemotherapy has been shown to induce growth arrest in AML cells and increase intracellular oxidized nucleotides, resulting in significant DNA damage. This combination therapy may extend the survival of AML patients (Karsten, 2022).

The recent advancements in cancer research have brought to light the significant roles of the MTH1 and PMS2 genes. PMS2, a dimerization partner of MLH1 and an essential component of the mismatch repair system, has been found to contain promoter mutations in approximately 10% of melanoma cases. These mutations correlate with a tumor mutation load more than five times higher than in tumors with wild-type PMS2 (Chalmers et al., 2017), The PMS2 and MTH1/NUDT1 genes are closely located on chromosome 7 (at 7p22.1 and 7p22.3, respectively). Experimental evidence from clinical histology suggests that simultaneous silencing of both MTH1 and PMS2 significantly induces cell death, highlighting a potential therapeutic approach (Tan et al., 2021; Das et al., 2022).

Furthermore, MTH1 has been identified as a tumor-promoting factor in various cancers. Its inhibition leads to an accumulation of reactive oxygen species (ROS) and subsequent DNA damage in tumor cells, resulting in varying degrees of antitumor effects. MTH1 inhibition has also been found to synergize with some anticancer drugs in many studies, enhancing their effectiveness in inhibiting tumor growth, migration, and invasion. This synergy has spurred increased research interest in MTH1, leading to the development and exploration of various MTH1 inhibitors and related therapeutic approaches.

## 5 Therapeutic programs related to MTH1

The development of antitumor drugs has increasingly focused on targeting key molecules in DNA damage repair pathways. A significant milestone in this field was the approval of the polyadenosine diphosphate ribose polymerase (PARP) inhibitor olaparib by the U.S. FDA in 2014. Olaparib, an antitumor drug that targets DNA damage repair, exemplifies the progressive focus on such therapies (Dominissini and He, 2014).

MTH1, due to its role in maintaining the stability of damaged genes by counteracting the stress damage produced by reactive oxygen species (ROS), has become a subject of interest in cancer research. This has led to the development of various MTH1 inhibitors and new inhibitor-based therapeutic modalities (Table 1). The mechanism involves redox regulation causing cells to produce ROS, leading to DNA damage and free deoxyribonucleotide triphosphates (dNTPs). MTH1 plays a critical role by eliminating excess dNTPs, preventing the incorporation of damaged bases during DNA replication. Notably, MTH1 is expressed more strongly in tumor cells compared to normal cells, making it a key target in cancer therapy.

**TABLE 1:** MTH1 inhibitors related to MTH1 and their functions.

Series	Inhibitors	IC50	Function	References
Small-molecule drugs	SCH5 1344	410 nmol/L	suppress the growth of RAS-overexpressing fibroblasts	Chalmers et al. (2017)
	crizotinib	1.4–4.3 μmol/L	Inhibit HGF/MET signaling and RhoA activation	Huber et al. (2014), Takiguchi et al. (2017)
	(S)-crizotinib	330 nmol/L	increase intracellular ROS levels	van der Waals et al. (2019), Li et al. (2021b)
Natural products	echinacoside	7 μmol/L	increase the cellular level of oxidative damage	Dong et al. (2015)
	farnesyl phenolic, FP1-8	8.6–28.5 μmol/L	bind to MTH1 within intact cells	Gao et al. (2017)
	α-mangostin	0.47 μmol/L	binds to the active site of MTH1	Yokoyama et al. (2019)
	γ-mangostin	2.0 μmol/L	N	Yokoyama et al. (2019)
	3-isomangostin	52 nmol/L	N	Yokoyama et al. (2019)
Pyrimidine analogues	TH287	0.9 nmol/L	cause the accumulation of oxidized dNTPs and cytotoxicity in cancer cells	Gad et al. (2014), Lee et al. (2019), Shi et al. (2019), Huang et al. (2020), Zhan et al. (2020)
	TH588	7.0 nmol/L	activates the mitotic surveillance pathway and thus prevents cancer cells from re-entering the cell cycle	Baloghová and Beninghaus (1985), Ikejiri et al. (2018), van der Waals et al. (2019)
	TH1579 (karonudib)	N	cause accumulation of oxidative lesions into DNA in an MTH1-dependent manner	Warpman Berglund et al. (2016), Hua et al. (2019), Moukengue et al. (2020), Hu et al. (2021), Sanjiv et al. (2021), Centio et al. (2022)
	aminopyrimidine (AP) analogue, AP1-12	0.2nmol/L-4μmol/L	bind to MTH1	Petrocchi et al. (2016)
	MI-743	91.44 nmol/L	induce accumulation of 8-oxo-dG lesions, DNA damage, and apoptosis	Zhou et al. (2019)
	MI-401	461.32 nmol/L		Zhou et al. (2019)
Purine analogues	P-1	2.8 μmol/L	inhibit MTH1	Kettle et al. (2016)
	purinone macrocycle (PM) analogue, PM-1	0.3 nmol/L	increase in potency against MTH1	Kettle et al. (2016)
	MTH1 binding site ligand fragment (F),1-5	6–79 μmol/L	bind to the MTH1 site and inhibits MTH1 activity	Rudling et al. (2017)
	purine (Pu) derivatives, PU1-8	3.3 mmol/L	inhibit MTH1	Kumar et al. (2017)
Quinoline and quinazoline analogues	3-amidoquinolines (AQ)	AQ-1 (0.9 nmol/L) and AQ-2 (12.5 nmol/L)	against MTH1 with high potency	Zhou et al. (2019)
	2-aminoquinazoline (AZ)	AZ-1 (3.32 mmol/L)	inhibit MTH1	Zhou et al. (2019)
Indole and indazole analogues	7-azaindole (AI), AI1-15	AI-2 (IC50 = 5 nmol/L)	inhibit MTH1	Rahm et al. (2018)
	7-azaindazole (AID) analogues, SB2001	3.57 mmol/L	bind and inhibit MTH1 and LTA4H, induce the accumulation of oxidative damages in DNA mismatch repair	Park and Park (2019)
Nucleotide analogues	8-halogenated 7-deazadGTP analogues	7deazadGTP (1.57 μmol/L); 7-I-7-deazadGTP (2.62 μmol/L)	seldom be hydrolyzed but obviously inhibited MTH1 activity	Yin et al. (2016a), Yin et al. (2016b)
Metalated analogues	Cd (II) and Cu (II)	Cd (II) (30 μmol/L) and Cu (II) (30 μmol/L)	N	Kasprzak and Bialkowski (2000)
	cyclometalated ruthenium (CR)	low-nanomolar levels	confirmed by testing with a large panel of protein kinases and other ATP binding proteins	Streib et al. (2014)

*N, no relevant studies available.

### 5.1 TH287 and TH588

The research by Gad et al. (2017) highlights the potential of targeting MTH1 in cancer treatment. In their study, they used small interfering RNA (siRNA) technology to knock down MTH1 in human osteosarcoma U2OS cells and normal VH60 cells. They observed that MTH1 played a more crucial role in the development of U2OS cells compared to VH60 cells. This finding led them to screen for inhibitory compounds using purified MTH1 protein, resulting in the identification of TH287 as an effective inhibitor with an IC50 value of 0.8 nmol/L. However, TH287 had poor stability, leading to the development of a modified compound, TH588, which not only retained inhibitory activity but also demonstrated improved stability and selectivity.

Interestingly, both TH287 and TH588 were found to selectively kill a variety of tumor cells without adversely affecting normal cells. *In vivo* experiments showed that TH588 inhibited the growth of subcutaneously transplanted melanoma by overcoming tumor resistance to carboplatin, azelnimidamide, and verofenib. Mechanistically, TH287 and TH588 function by occupying the binding pocket of 8-oxo-dGTP in MTH1, interacting with key amino acid residues in the target protein.

In the realm of gastric cancer research, TH287 has shown promising results. It impacts mitochondrial function and inhibits the PI3K/AKT signaling pathway, playing a significant role against gastric cancer. This highlights TH287’s potential as a valuable tool in the treatment of gastric cancer by targeting specific molecular pathways (Zhan et al., 2020). Additionally, TH588, which is not only an MTH1 inhibitor but also a microtubule-targeting agent, demonstrates its anticancer efficacy by inhibiting microtubule polymerization and reducing microtubule movement during cell division. This action effectively delays the process of cell division, contributing to its anticancer properties (Rajendraprasad et al., 2021). Both TH287 and TH588 have progressed to clinical studies, marking a significant step forward in the development of these compounds as potential cancer therapies. This advancement into clinical trials underscores the potential of these drugs in providing new treatment options for cancer patients, especially in targeting specific molecular mechanisms involved in cancer progression (Gad et al., 2014).

### 5.2 TH1579 (Karonudib)

The development of TH1579 (Karonudib), an optimized analog of TH588, marks a significant advancement in cancer therapeutics. Currently in phase I clinical trials, TH1579 is being tested for its efficacy in treating both solid and hematologic cancers. These trials represent a crucial step in evaluating the drug’s safety and effectiveness in a clinical setting (Moukengue et al., 2020).

TH1579 operates by a mechanism similar to TH588, showcasing high selectivity in targeting cancer cells. It blocks cellular mitosis and activates the spindle assembly checkpoint (SAC), leading to an increase in reactive oxygen species (ROS). The elevated ROS levels oxidize the dGTP pool, resulting in an excess of 8-oxo-dGTP. This oxidized nucleotide is then incorporated into DNA during mitotic replication, effectively targeting and killing cancer cells. Additionally, TH1579 has shown promising results when used in combination with BRAF inhibitors, particularly in cells with BRAF mutations. This combination therapy has exhibited enhanced cell-killing effects both *in vivo* and *ex vivo*, suggesting a potential synergistic approach in cancer treatment (Das et al., 2020).

In terms of immunotherapy, TH1579 does not appear to compromise the antitumor responses induced by anti-CTLA-4 antibody treatment in melanoma. Furthermore, it has been observed that Karonudib does not alter the tumor suppressor activity produced by the injection of autologous tumor-infiltrating lymphocytes (TILs) in a patient-derived xenograft (PDX) model of melanoma. This implies that TH1579 (Karonudib) does not interfere with T-cell-mediated cytotoxicity *in vivo*, an important consideration for combination therapies involving immunotherapeutic agents (Einarsdottir et al., 2018).

Overall, the development and clinical trials of TH1579 (Karonudib) represent an exciting advancement in the field of oncology, potentially offering new therapeutic options for patients with various forms of cancer.

### 5.3 Crizotinib

Mutations in RAS isoforms are a common occurrence in human cancers (Moore et al., 2020). The study by Huber et al. sheds light on the role of MTH1, a human mut-T homologue, in the context of these mutations, particularly in KRAS tumor cells. Their research revealed that loss of MTH1 function impairs the growth of KRAS tumor cells, while overexpression of MTH1 reduces sensitivity to the compound SCH51344 (Huber et al., 2014).

SCH51344, though not used clinically, led to the identification of crizotinib, a widely used dual Met/ALK inhibitor, as a potent inhibitor of MTH1 activity. Interestingly, while (R)-crizotinib, the clinically used form, is inactive against MTH1, the (S)-enantiomer of crizotinib selectively inhibits MTH1 catalytic activity. This selective inhibition was demonstrated to have a significant effect on human tumor cells by inducing DNA damage *in vitro* and *in vivo* experiments, without significantly impacting normal cells. The mechanism behind the selective action of (S)-crizotinib involves the formation of hydrogen bonds with fewer amino acid residues in the MTH1 active pocket. This reduced activity contributes to its specificity and effectiveness in targeting tumor cells harboring RAS mutations, offering a potential therapeutic strategy for cancers driven by these mutations (Huber et al., 2014).

These findings highlight the potential of targeting specific molecular pathways in cancer therapy, particularly those involving key enzymes like MTH1, and open up avenues for the development of more effective and selective cancer treatments.

### 5.4 Combination of MTH1 inhibitors with new therapeutic regimens

The development of MTH1 inhibitors in recent years has largely focused on their potential to kill tumor cells, supporting the idea of MTH1 inhibition as an effective strategy for tumor suppression. However, some studies have drawn opposite conclusions, particularly regarding second-generation inhibitors (Huber et al., 2014). For instance, research by Govindi et al. highlighted a complex scenario where certain cancer cell lines, possessing 8-oxo GTPase activity, appeared almost completely independent of MTH1. These cell lines were not affected by MTH1 depletion or MTH1 inhibitors. Interestingly, the induction of cytotoxicity by TH588 and TH287 was still significant in these cell lines. This suggests that MTH1 inhibition might still play a role in impacting biological functions, albeit in a more nuanced manner than previously thought (Samaranayake et al., 2020).

Given these findings, researchers are now exploring the combination of first-generation MTH1 inhibitors with other therapeutic approaches. They are also developing new treatment protocols that integrate MTH1 inhibitors with different therapeutic methods. This approach aims to maximize the potential benefits of MTH1 inhibition while mitigating any limitations observed in certain cancer types or with specific inhibitors.

This evolving research landscape underscores the importance of continued investigation into the role of MTH1 in cancer and the potential for varied responses to its inhibition across different cancer cell types. It highlights the need for a more tailored approach to cancer therapy, taking into account the specific characteristics and vulnerabilities of different tumor cells.

The combination of the MTH1 inhibitor TH1579 (Karonudib) with radiation therapy (RT) represents a significant advancement in cancer treatment strategies. This combination has been shown to increase the sensitivity of cancer cells to radiotherapy. It also counteracts the resistance to radiotherapy often induced by hypoxic stress in tumors. This finding is a crucial development, as overcoming hypoxia-induced resistance can significantly improve the efficacy of radiation therapy in cancer treatment. Additionally, the use of piperlongumine (PLN) has been found to disrupt redox homeostasis, thereby increasing the sensitivity of cancer cells to MTH1 inhibitors. This approach demonstrates the potential of targeting redox balance in cancer cells as a way to enhance the effectiveness of MTH1 inhibition (Hansel et al., 2021).

Photodynamic therapy (PDT) is another promising strategy for tumor treatment. It works by generating highly toxic reactive oxygen species (ROS) that damage DNA and other essential biomolecules. However, the effectiveness of PDT can be limited due to local hypoxia within tumors and the presence of ROS defense mechanisms like MTH1, which can counteract the damage caused by ROS-oxidation. Innovatively, Son et al. (2022) developed a nanoplatform that leverages these mechanisms to enhance PDT’s effectiveness. They enabled the *in-situ* growth of platinum nanoparticles (Pt NPs) with peroxidase activity in the nano-channels of mesoporous silica nanoparticles (MSNs). By encapsulating chlorinated photosensitizer e6 (Ce6) and the MTH1 inhibitor TH588, and further modifying them with arginine-glycine-aspartic acid (RGD)-functionalized liposomal shells, they created the MPCT@Li-R NPs. These nanoparticles can continuously catalyze the decomposition of hydrogen peroxide (H_2_O_2_) to oxygen (O_2_) in tumors, thereby promoting the generation of singlet oxygen during PDT and improving the oxidative damage to DNA bases. The acid-responsive release of TH588 inhibits MTH1-mediated scavenging of oxidized bases, further aggravating oxidative DNA damage. This innovative approach enhances the performance of PDT in hypoxic tumors, suggesting the potential for effective cancer cell killing without generating excess cytotoxic ROS (Zhao et al., 2020).

In the field of enhancing photodynamic therapy (PDT), researchers like Chen et al. and Shao et al. have made significant strides with their innovative approaches. Chen et al. (2023) developed a novel nanoamplifier, termed IrP-T, which leverages the properties of an amphiphilic iridium complex and an MTH1 inhibitor (TH287). This nanoamplifier is designed to inflict ROS-induced damage when activated by light. It works by increasing the accumulation of 8-oxo-dGTP via the MTH1 inhibitor, thereby enhancing oxidative stress and inducing cell death. This approach effectively capitalizes on the oxidative damage mechanism to boost the therapeutic efficacy of PDT.

Similarly, Shao et al. (2023) devised a hypoxia-activated nanosystem named FTPA. This innovative system can degrade under hypoxic conditions to release its encapsulated components: a PDT photosensitizer named 4-DCF-MPYM, and the MTH1 inhibitor, TH588. Upon activation, this nanosystem exerts a potent antitumor effect. This strategy is particularly ingenious as it targets the hypoxic environment of tumors, which is a common challenge in effective cancer treatment.

These studies represent a significant advancement in cancer therapy, particularly in the utilization of nanotechnology to enhance the efficacy of PDT. They showcase the potential of combining innovative drug delivery systems with targeted therapy to overcome some of the traditional limitations in treating cancer.

## 6 Conclusion and perspective

The role of redox homeostasis disturbances in disease development, particularly in the context of cancer, is increasingly recognized. High levels of reactive oxygen species (ROS) in cells can directly affect DNA biological processes, leading to DNA damage. MTH1 plays a critical role in counteracting such DNA damage and maintaining the integrity of DNA, which is crucial for normal cell metabolism. In contrast, tumor cells rely heavily on MTH1 to support their distinctive biological properties.

Normal cells, which do not typically experience redox imbalance during metabolism, do not depend on MTH1 function for maintaining growth. However, tumor cells often face various external stimuli that cause DNA damage. MTH1 repairs this damage, contributing to the survival and proliferation of tumor cells. Therefore, MTH1 emerges as a potential target for cancer treatment and intervention. Several approaches to target MTH1 for tumor intervention and treatment have been considered:

1. *Targeting MTH1 Protein Function:* Directly applying MTH1 inhibitors to induce DNA damage, increase cytotoxicity, and promote apoptosis in tumor cells, thereby inhibiting tumor growth.
2. *Targeting MTH1 Transcription-Translation Process*: Developing inhibitors, such as non-coding RNAs, to inhibit MTH1 expression and its tumor-promoting effects.
3. *Targeting MTH1 Repair Process*: Inhibiting the repair process of DNA synthesis, promoting DNA damage, and inhibiting tumor growth.
4. *Utilizing ROS Release Factors:* Using factors that promote elevated ROS levels in conjunction with MTH1 inhibitors to enhance DNA damage in tumor cells.
5. *Promoting 8-oxo-dGTP Accumulation*: Researching and developing regulatory mechanisms that accelerate or amplify the effects of MTH1 inhibitors in promoting DNA damage in tumors.

The research surrounding MTH1 and its inhibitors is indeed still in its nascent stages, with most development concentrated in preclinical trials. This stage of research is crucial for understanding the efficacy and safety of these inhibitors before they can be considered for clinical use. The question of whether MTH1 can become a viable and effective target for the clinical treatment of tumors remains open and requires further empirical validation.

Ongoing research in biochemistry, genetics, oncogenes, and signal transduction is critical to uncovering the full potential of MTH1 as a therapeutic target. As our understanding of these areas deepens, the opportunity to explore MTH1’s activity in interfering with and treating tumor development becomes more promising. The complexity of tumor biology necessitates a comprehensive approach to treatment, and targeting specific proteins like MTH1 could offer a new pathway for effective cancer therapies.
